# Acute care of aneurysmal subarachnoid hemorrhage: practical consensus statement from a multidisciplinary group of German-speaking neurointensivists and neuroradiologists on behalf of the DIVI neurology section

**DOI:** 10.1186/s42466-025-00407-x

**Published:** 2025-07-30

**Authors:** Rainer Kollmar, Hagen B. Huttner, Yigit Ozpeynirci, Christian Herweh, Jochen A. Sembill, Stefan Gerner, Michael Bender, Patrick Schramm, Ingo Schirotzek, Lisa Maeder, Anisa Myftiu, Marius Hartmann, Juergen Konczalla, Karsten Geletneky, Rainer Kram, Raimund Helbok, Joji B. Kuramatsu, Martin Welte, Amr Abdulazim, Emanuela Keller, Ferdinand Bohmann, Wolf-Rüdiger Schäbitz

**Affiliations:** 1 Klinik für Neurologie und Neurointensivmedizin, Darmstadt, Germany; 2 Neurologische Universitätsklinik Erlangen, Friedrich-Alexander-Universität Erlangen Nürnberg, Klinikum Darmstadt, Grafenstrasse 9, 64283 Darmstadt, Germany; 3 Universitätsklinikum Carl Gustav Carus Dresden, Klinik für Neurologie, Dresden, Germany; 4 Department Neuroradiologie, Universitätsklinik Giessen, Giessen, Germany; 5 Department für Neuroradiologie, Universitätsklinikum Heidelberg, Heidelberg, Germany; 6 Klinik für Neurochirurgie am Universitätsklinikum Giessen und Marburg GmbH, Giessen, Germany; 7 Klinik für Neurologie am Universitätsklinikum Giessen und Marburg GmbH, Giessen, Germany; 8 Institut für Radiologie, Neuroradiologie und Nuklearmedizin, Klinikum Darmstadt, Darmstadt, Germany; 9 Klinik für Neurochirurgie, Goethe-Universität Frankfurt am Main, Frankfurt, Germany; 10 Institut für Neurochirurgie, Klinikum Darmstadt, Darmstadt, Germany; 11 Klinik für Anaesthesiologie, Universitätsklinikum Düsseldorf, Düsseldorf, Germany; 12 Universitätsklinik für Neurologie, Johannes Kepler Universität Linz, Linz, Austria; 13 Neurologische Klinik, RoMed Klinikum Rosenheim, Rosenheim, Germany; 14 Klinikum Darmstadt, Klinik für Anästhesie und operative Intensivmedizin, Darmstadt, Germany; 15 Klinik für Neurochirurgie, Universitätsklinikum Mannheim, Mannheim, Germany; 16 Institut für Intensivmedizin, Universitätsklinikum Zürich, Zürich, Switzerland; 17 Klinik für Neurologie, Goethe-Universität Frankfurt am Main, Frankfurt, Germany; 18 Universitätsklinik für NeurologieEvangelisches Klinikum BethelUniversitätsklinikum OWL, Universität Bielefeld, Bielefeld, Germany

## Abstract

**Background:**

Aneurysmal subarachnoid hemorrhage (aSAH) is a critical condition requiring multidisciplinary management, particularly in the intensive care setting. Despite existing guidelines, gaps in evidence and variability in practice remain, necessitating practical, consensus-driven recommendations for acute care and management.

**Objective:**

To develop comprehensive, practical consensus statement for the acute management of aSAH, addressing high- and low-evidence areas, through a modified Delphi consensus approach among German-speaking neurointensivists and neuroradiologists.

**Methods:**

Senior experts from neurology, neurosurgery, neurocritical care, and interventional neuroradiology were selected for their academic and clinical expertise. The consensus process included iterative rounds of Delphi surveys, a face-to-face meeting, and online discussions. Consensus statements were formulated based on literature review, expert input, and iterative validation, with a consensus threshold of ≥ 70% agreement.

**Results:**

The group reached consensus on key aspects of aSAH management, including diagnostic protocols, invasive monitoring, blood pressure and temperature control, prophylactic and therapeutic measures for vasospasm and delayed cerebral ischemia, nutrition, and mobilization. Specific guidance was provided for early surgical/endovascular intervention, invasive hemodynamic monitoring, enteral nutrition initiation, and fever prevention. The consensus emphasized evidence-informed strategies where available and expert-derived recommendations in areas lacking robust data, such as therapeutic hypothermia and multimodal monitoring.

**Conclusion:**

This practical consensus statement provides a standardized approach to aSAH management, balancing guideline-based evidence with expert consensus to address clinical uncertainties. Due to the used methods and composition of the group, the results should be considered as a multi-institutional protocol of an experienced neurointensivist group, but certainly not as evidence based-guidelines. Adoption of this consensus may improve outcomes and harmonize care in the intensive management of aSAH.

## Background

Spontaneous aneurysmal subarachnoid hemorrhage (SAH) is a life-threatening disease which requires multidisciplinary approaches for medical management [1]. Early diagnosis, appropriate and early treatment of the aneurysm, and postinterventional care are important issues to improve outcome. Many SAH-patients need intensive care treatment at least in the early stage of the disease. The period for intensive care takes often many days or even weeks due to different complications, such as vasospasm, delayed cerebral ischemia (DCI), infectious complications and drainage management [1, 2]. Although guidelines give reasonable advice for medical care after SAH, the management, especially in the ICU is often variable due to uncertainties within the guidelines and individual interpretation [1–3]. Recently, these knowledge gaps and topics for future research have been perfectly addressed in the latest guideline from the American Heart Association (AHA) [1]. For example, severe vasospasm and DCI appear frequently in severely affects SAH patients, but potentially promising treatment such as interventional spasmolysis are only level B recommendations due to non-randomized studies. However, the treating intensive care team must deal with both standard conditions and situations where evidence is limited despite frequent appearance. The practical approach for providing intensive care to severely affected SAH patients has been evaluated through an online survey that encompasses intensive care units in German-speaking countries [3]. This survey has revealed that most patients are being treated in accordance with the guidelines in numerous aspects. However, a remarkable number of centers have treated SAH patients similarly in situations where high levels of evidence are missing. For example, over 50% of respondents used interventional techniques to treat vasospasm. Furthermore, targeted temperature management is often used to prevent fever and induced hypothermia to protect the brain [3]. At present, German and European Guidelines for the management of aSAH are outdated or do not address specific intensive care topics. Based on these findings and knowledge gaps identified and described in recent guidelines, a group of specialists in the acute care was asked on behalf of the German Interdisciplinary Association for Intensive Care and Emergency Medicine (Deutsche Interdisziplinäre Vereinigung für Intensiv- und Notfallmedizin/DIVI) neurological section named “studies and standards” to work out practical consensus statements for the acute treatment of SAH including the intensive care phase and topics with lower level of evidence. These practical consensus statement could be used [1] as a basis for intensive care directed German or European recommendations or/and [2] practical in-house implementation. The character of this consensus statement could help to prepare and roll out harmonized protocols to facilitate the planned SAH registry within the DIVI neurology section The group was asked to use a modified Delphi consensus approach for this topic.

## Methods

### Selection of participants

Senior physicians who were considered to have an academic and practical expertise in the treatment of acute SAH were contacted by the speaker of the DIVI section neurology to participate the consensus group. The group consisted of neurologists (n = 11), neurosurgeons (n = 4), neuroradiologists (n = 3), and intensivists (n = 4). All neurologists, neurosurgeons and intensivists had to be board certified in their medical speciality (neurology, neurosurgery, anaesthesiology) and intensive care medicine. Radiologists had to be board certified for radiology and neuroradiology including the highest level certification for interventional neuroradiology in Germany. All participants are employed in medical institutions with a mean treatment rate of more than 50 aSAH per year.

### Delphi consensus

A modified Delphi consensus approach was used, which involved a combination of online questionnaires, a face-to-face meeting, and post-meeting reviews.

- The initial questions were formulated by a core group and modified by all participants of the group before the first face-to-face meeting.
- The first face-to-face meeting took place in summer 2023. The process consisted of two rounds of a Delphi questionnaire in 2023. Questions were rephrased by the group, if necessary, followed by voting. The agreed cut-off of for the consensus was 70% of experts in agreement; this was in keeping with recent consensus initiatives in this field [4, 5]. Where consensus (≥ 70% agreement) was achieved, the discussion focused on improvements in the phrasing or scope of the initial statement to arrive at a final statement that clearly captured the consensus views of all experts. Where consensus was not reached, a detailed facilitated discussion was undertaken to identify the reasons for the lack of agreement and documented.
- The responses and voting of the meeting were captured in a summary document that showed how the consensus evolved at the meeting. Following the face-to-face meeting, each question has been attributed to a group of 3 participants to formulate the final text of the consensus statement. Statements and questions were informed by a literature search; the search yielded very few publications relevant to the specific topics under discussion.
- This summary was distributed to all participants via e-mail and additional comments from all participants of the group were collected.
- For round 3, areas requiring additional discussion were identified, and the process for addressing these was guided by the chair of the meeting. Round 3 was held as an online meeting.
- Based on the final discussion in round 3, a manuscript was created, structuring the consensus statement, adding additional narrative, and providing context. This manuscript was distributed to all participants for review and final validation.

### Consensus statements

**Fig. 1:**
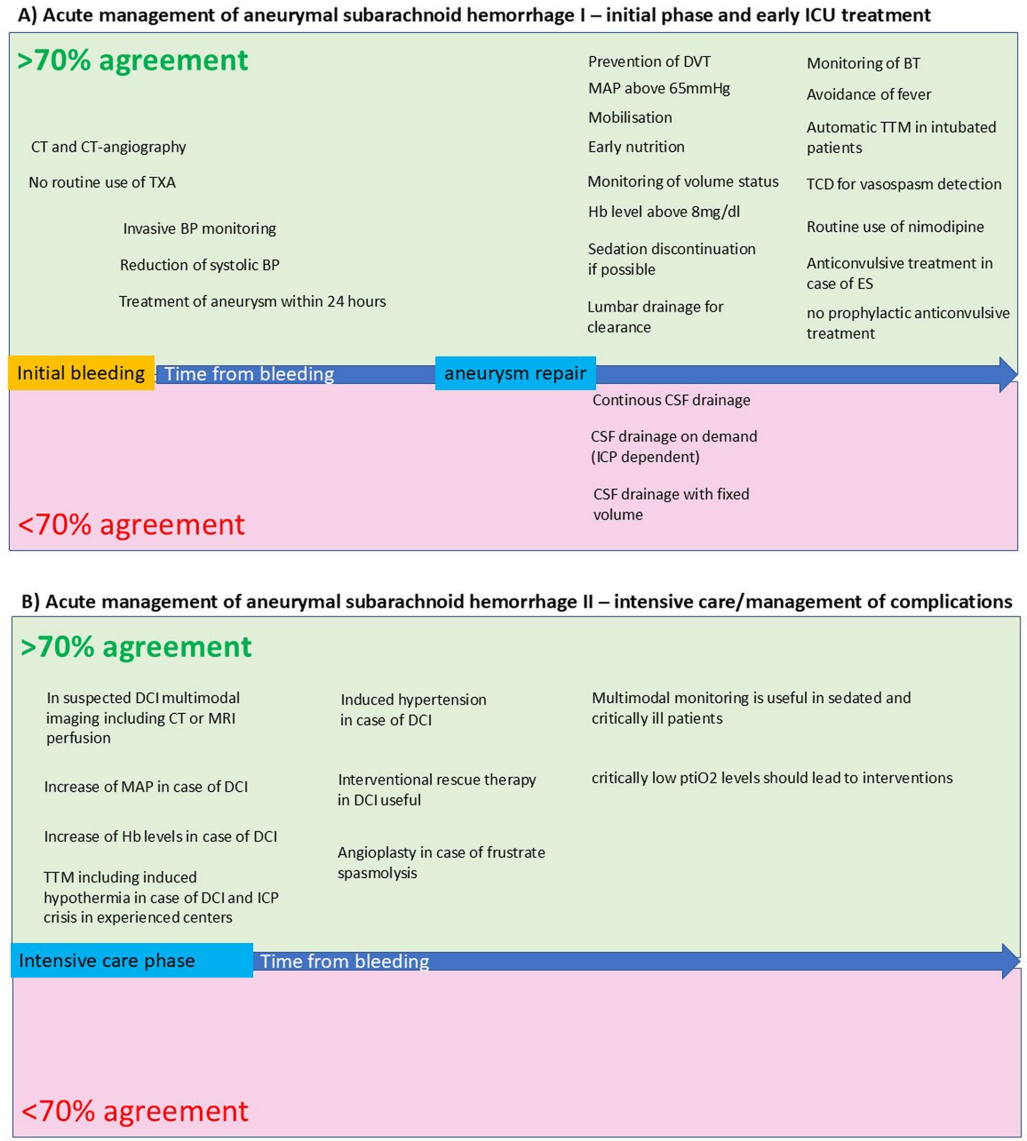
Visual presentation of consensus statements. **A** initial phase of SAH and early ICU treatment and **B** the phase of intensive care and management of complications. The blue arrow in the middle of **A** and **B** represents the time from bleeding (left side) into the following period of aneurysm repair and ICU treatment (right side). The green box indicates fields in which we reached an over 70 % agreement, while the red box shows agreement below 70%

The sequence of consensus statements follow the timing of care for patients with acute aSAH. To provide a more concise overview, in addition to the subsequent text, a visual presentation of these consensus statements has been provided in Fig. 1 of this manuscript.

#### Management before treatment of the aneurysm

1. Tranexamic acid should not be used routinely in aSAH to prevent rebleeding.

100% agreement.

Rebleeding is a prevalent complication of SAH, with a maximum risk of 4 to 20% within the initial 24 h [6]. Therefore, the early application of antifibrinolytic agents, such as tranexamic acid (TXA), is a promising strategy. Nonetheless, the data regarding the prevention of rebleeding by TXA are inconsistent across trials [1, 7, 8]. Moreover, TXA does not improve the functional outcome after SAH.

“Consensus statement: Our expert panel does not recommend the routine use of tranexamic acid in SAH”.

2. CT-Angiography should be included in the initial diagnostic procedure after the diagnosis of SAB.

87.5% agreement (2 abstentions, 2 votes against).

The current guidelines from AHA/ASA and recent reviews suggest performing vascular imaging when a spontaneous SAH is diagnosed [1, 12, 13]. Vascular imaging should be done if it exhibits a typical pattern, such as diffuse basal cistern or sylvian fissure SAH, on non-contrast CT scans. As CT-angiography (CT-A) is commonly available and often the primary diagnostic test following the identification of aSAH on non-contrast CT, CT-A is useful for choosing the appropriate treatment method (endovascular or neurosurgical). An alternative to CT-A for detecting aneurysms is Magnetic Resonance Angiography (MRA). However, MR-A’s sensitivity is limited, and it has drawbacks related to accessibility and acquisition time.

“Consensus statement: Our expert panel recommends that CT-A should be conducted for every patient diagnosed with aSAH on CT scans to get information about the configuration of the aneurysm”.

3. Blood pressure should be measured invasively in patients with aSAH.

100% agreement.

Current AHA/ASA guidelines recommend frequent blood pressure monitoring in aSAH patients [1]. The authors here recommend that invasive monitoring of arterial blood pressure should be preferred over noninvasive blood pressure monitoring to provide valid and continuous values for adjusting and monitoring hemodynamic therapy. In addition, an arterial cannula offers a simple way of closely monitoring blood glucose levels and the option of pulse contour analysis. However, there is no valid evidence that one method or the other has a more favorable impact on patient outcomes.

“Consensus statement: Our expert panel recommends the routine use of invasive blood pressure monitoring”.

4. The systolic blood pressure should be lowered to a target range of at least 160 mmHg systolic after the diagnosis of aSAH and before the source of bleeding is treated.

100% agreement.

The importance of managing the hemodynamic situation of patients prior to the treatment of aneurysmal SAH is still unclear [1, 2]. In principle, there is a risk that too low systolic blood pressure (BP) increases the occurence of DCI and too high blood pressure increases the risk of rebleeding. Based on a meta-analysis, a systolic blood pressure above 160 mmHg suggested higher re-bleeding rates [1]. However, as stated in the recent AHA guideline, the available evidenceis insufficient to recommend any specific BP target. The lower systolic BP Level is as well unclear and may be targeted in a range between 100 – 120 mm Hg BP [1, 14]. In a retrospective cohort study including 522 SAH patients in two neurological units, lood Pressure below 140 mm Hg versus treatment of the blood pressure in “extreme Situations” showed no significant difference in the risk-adjusted analysis [15]. Until valid prospective data are available, only low evidence and a high level of recommendations can be made.

“Consensus statement: Our expert panel recommends lowering blood pressure below 160mmHg systolic before the aneurysm is treated despite low level of evidence”.

5. For patients with aSAH, surgical or endovascular treatment of the ruptured aneurysm should be performed as early as feasible after presentation, preferably within 24 h of onset, to improve outcome.

100% agreement

Early treatment of ruptured aneurysms reduces the risk of rebleeding and facilitates treatment of DCI. Therefore, guidelines recommend early interventional or surgical treatment of the aneurysm based on various studies including the randomized ISAT trial (International Subarachnoid Aneurysm Trial) [1, 9]. Meta-analyses support the outcome benefit of early treatment including in patients with high-grade aSAH [8]. The data demonstrate a beneficial effect of treatment < 24 h versus > 24 h from ictus but have not been able to similarly demonstrate significant beneficial difference between < 24 h and 24 to 72 h [1, 10, 11].

“Consensus statement: Our expert panel recommends early surgical or endovascular treatment within 24 hours of onset”.

### General intensive care measures after aneurysm repair

6. The mean arterial pressure (MAP) should be set to a value of at least ≥ 65 mm Hg after aSAH treatment.

100% agreement.

An excessive reduction in arterial blood pressure following treatment of an aneurysmal SAH can lead to a reduced oxygen and glucose supply to all organ systems. Previous data suggests that high variations in blood pressure worsen outcome [16]. To ensure adequate perfusion, a mean arterial blood pressure of over 65 mmHg should be selected. This limit must of course be exceeded in the event of delayed cerebral ischemia (DCI), increased intracranial pressure or renal dysfunction. Surviving Sepsis Campaign guidelines recommend an initial MAP target value of 65 mmHg in patients with septic shock [17].

“Consensus statement: Our expert panel recommends a level of above 65mmHg mean arterial pressure after aneurysm repair despite low level of evidence.”

7. Patients with aSAH should be treated pharmacologically or by intermittent pneumatic compression for prevention of deep venous thrombosis within 24 h after SAH.

100% agreement.

Patients with nontraumatic SAH often have a prolonged hospital course. This may increase the risk for venous thromboembolism with approx. 4–24% (VTE).[1, 18, 19] One small RCT with enoxaparin once daily in aSAH after aneurysm treatment found that enoxaparin did not increase bleeding and may have decreased the VTE rate.[18] The data on the timing of thrombosis prophylaxis is poor and is based primarily on retrospective studies.[19, 20] A case–control study comparing early (≤ 24 h after aneurysm occlusion) with delayed (> 24 h) pharmacological prophylaxis showed no intracranial hemorrhagic complications in the early group.[21]. There are few data concerning the effectiveness of intermittent pneumatic compression (IPC) in treating patients with stroke The largest clinical study on IPC and VTE in acute neurovascular injured patients excluded SAH [22], but found IPC being an effective and inexpensive method of reducing the risk of DVT and improving survival in immobile stroke patients. In a retrospective monocentric analysis of SAH patients including correct and early use of intermittent pneumatic compression and subcutaneous heparin, this bundle care did not affect the incidence of VTE [23].

“Consensus statement: Our expert panel recommends the prevention of deep venous thrombosis within 24 hours after SAH with pharmacological means such as heparinoids or/and intermittend pneumatic compression.”

8. Hemodynamically stable patients should be mobilized early after aneurysm treatment according to a standardized protocol if they remain neurologically unchanged.

88% agreement (2 abstentions)

Immobility in stroke patients contributes to numerous secondary problems such as thromboembolism, pressure ulcers, pneumonia and an early mobilization strategy may enhance functional recovery after 90 days [1]. A recent systematic review and meta-analysis on early mobilization included 1,1757 patients and showed improved outcome at discharge and at three months [24]. Moreover, early mobilization was associated with a reduction in radiological and clinical cerebral vasospasm rate, although mechanisms of these effects are not clear and highly spectulative.. Adverse events related to early mobilization were up to 6% and included mostly hemodynamic instability.

6% of mobilization sessions involved adverse events, mostly haemodynamic changes.

All studies included in the recent meta-analysis defined monitoring parameters and criteria for stopping mobilization mostly including haemodynamic and respiratory instability, an increase of intracranial pressure (ICP), acute change in neurological examination, and signs of intolerance such as nausea.

“Consensus statement: Our expert panel recommends early mobilization after SAH, when the patient is in stable conditions and has no increased ICP. As described in the recent paper, a standardized local standard operating procedure (SOP) should be available with defined starting and stopping criteria”.

9. Enteral nutrition should be started at day one after aneurysm treatment.

100% agreement

Early enteral nutrition (EEN) (< 48 h) in mechanically ventilated patients has demonstrated reduction in complications, shortened hospital stay, and improved discharge prognosis [25]. Additionally, EEN may attenuate the inflammatory response of the brain-gut axis, thereby reducing the incidence of chronic hydrocephalus [26]. However, data specifically targeting stroke or especially SAH-patients are scarce. In one single-center retrospective study involving patients with aneurysmal subarachnoid hemorrhage who underwent craniotomy and aneurysm clipping alongside enteral nutrition, EEN with high-protein whey peptide digestion nutrients was associated with improved modified Rankin Scale (mRS) scores at discharge and reduced incidence of diarrhea in subarachnoid hemorrhage patients, suggesting that nutrient selection may influence outcome and prognosis [26]. High-protein nutrition in the acute phase of subarachnoid hemorrhage contributes to temporal muscle preservation and enhances oral intake [27]. In summary, specific nutritional guidelines tailored for subarachnoid hemorrhage (SAH) patients are lacking, implying that prevailing protocols established for general intensive care unit (ICU) patients, as outlined in recognized nutrition guidelines such as those by ESPEN, can be adopted.

“Consensus statement: Our expert panel recommends early enteral nutrition at day one after aneurysm repair according to protocols established for general intensive care unit.”

10. In patients with aSAH monitoring of volume status and aiming for euvolemia is reasonable.

100% agreement.

Hypovolemia following aneurysmal subarachnoid hemorrhage (SAH) increases the risk for delayed cerebral ischemia (DCI) [28, 29]. The most effective approach for evaluating and continuously monitoring intravascular volume status and fluid responsiveness in critically ill patients, including those with aSAH, remains controversial. Substantial evidence indicates that central venous pressure poorly correlates with circulating blood volume and lacks the capability to forecast hemodynamic response to a fluid challenge in critically ill patients. Consequently, central venous pressure seems to be an inadequate surrogate measure for intravascular volume status [30]. Continuous monitoring and optimization of hemodynamic parameters should guide fluid and hemodynamic management in aSAH patients during intensive care unit (ICU) treatment. Thus, parameters such as cardiac output, preload, and stroke volume variability can aid in identifying dehydration and promptly addressing it. Particularly for individuals with poor-grade aSAH, studies have demonstrated the advantages of early goal-directed treatment in reducing the incidence of DCI and improving functional outcomes at three months [31–33]. Therefore, the authors recommend considering invasive hemodynamic monitoring (e.g., PiCCO system) in patients with poor-grade SAH (WFNS IV-V) or hemodynamically unstable patients.

“Consensus statement: Our expert panel recommends monitoring of the volume status and euvolemia as a goal.”

11. In patients with aneurysmatic hemorrhage hemoglobin level is reasonable to be maintained ≥ 8 mg/dl.

100% agreement.

Anemia is common and associated with poor aSAH outcome. The German, European, and AHA/ASA guidelines give no official recommendation regarding erythrocyte replacement and the target hemoglobin (Hb) value. Some studies showed that Hb levels < 11.1 g/dl were associated with a poor outcome [34]. In addition, Hb-levels < 9 g/dl showed an increased incidence of cerebral hypoxia and dysfunction in cell energy (defined as lactate/pyruvate ratio > 40 in microdialysis) in patients with poor-grade SAH [35]. The recently published SAHaRA study investigated a liberal strategy (mandatory transfusion at a hemoglobin level of ≤ 10 g per deciliter) vs. a restrictive strategy (optional transfusion at a hemoglobin level of ≤ 8 g per deciliter) in critically ill patients with acute SAH [36]. There was no significant difference in the neurological outcome after 12 months, while absolute numbers indicated a trend towards better neurological outcome in liberal strategy. In conclusion there is a lack of reliable evidence for the benefit of higher hemoglobin levels, and therefore no strong recommendations and target values can be given. However, based on available studies and pathophysiological mechanisms in DCI and vasospasm, the practical advice of the consensus group is to define a specific Hb level.

“Consensus statement: Our expert group recommends transfusion when Hb levels are below 8g/dl.”

12. In the case of DCI, higher hemoglobin levels can be aimed for on an individual basis.

100% agreement.

Anemia is frequent (about 30%) among patients with aSAH and associated with poor neurological outcome [1]. In the case of DCI, anemia can contribute to ischemia from a pathophysiological point of view. One small RCT of 44 aSAH patients investigated a target hemoglobin concentration of at least 10 vs. 11.5 g/dl, which was safe and feasible without effects on clinical outcomes in underpowered analyses [35]. One retrospective analysis suggested that transfusion of anemic patients did not improve long-term outcome or mortality rates, but transfusion of patients with a hemoglobin concentration > 10 g/dL was associated with improved neurological outcomes, with no differences in mortality rates [34]. There is thus a lack of reliable evidence for the benefit of higher hemoglobin levels, which is why no strong recommendations and target values can be given. Nevertheless, in the opinion of the authors, a higher hemoglobin value can be aimed for in individual cases and in the situation of DCI.

“Consensus statement: Our expert group recommends higher Hb levels than 8g/dl in case of DCI based on individual decision.”

13. For continuous neurological assessment, sedation should be reduced or discontinued after aneurysm repair in all patients without contraindications.

100% agreement.

Continuous neurological assessment is a major part of daily intensive care unit treatment to detect neurological deterioration in an early stage. However, there are no recommendations, in neither AHA/ASA guidelines nor German guidelines for sedation management after aneurysm treatment. Measurement of ICP as well as multimodal cerebral monitoring is affected by not negligible limitations. In addition, no data are available, which demonstrate a benefit of sedation including ICP-measurement or multimodal monitoring compared to a clinical assessment in awake SAH patients. In recent years and promoted by the current German DAS-Guidelines, a more symptom-oriented therapy approach in critical care sedation management has been emphasized, avoiding deep sedation as far as possible except for specific indications. Therefore, the members of the DIVI study group consented on an early reduction or stop of sedation, if there are no contraindication (e.g. severe ARDS, raised ICP) to improve neurological assessment and prematurely recognize new neurological deficits.

“Consensus statement: Our expert group recommends reduction of sedation for frequent neurological clinical assessment in patients without contraindications.”

### Temperature management

TTM is a potential neuroprotective method in critically acute brain injury patients. While evidence for TTM including therapeutic hypothermia is low in SAH patients, our previously published survey on intensive care in German speaking countries indicates frequent use of TTM for aSAH patients in high volume centers [3]. Therefore, we gave this topic more space for discussion and consensus statements than was supported by high-level evidence.

14. Body core temperature should be monitored continuously in the acute phase after aSAH.

100% agreement.

Fever is frequently observed after SAH [40]. It is associated with DCI and vasospasm, resulting in a decreased functional outcome and a higher mortality rate following SAH [41–44]. Recording temperatures is crucial for treating and avoiding a fever. The group agreed that temperature should be monitored closely in patients with aSAH. Continuous measurement was the preferred option for all patients, but at least an hourly measurement was recommended by the group to avoid missing large fluctuations in temperature. Since brain temperature measurement is usually not done due to technical reasons or a lack of indication for brain probes, body core temperature should be measured as the most useful surrogate measure of brain temperature [45, 46]. Oesophageal or bladder probes are recommended for temperature measurement during Targeted Temperature Management (TTM) after SAH. This is consistent with the guidelines of the US Neurocritical Care Society (NCS) [47]. An hourly tympanic temperature measurement should be done for patients who do not need a urinary catheter.

“Consensus statement: Our expert group recommends continuous monitoring of body core temperature.”

15. Fever should be avoided in patients with acute SAH.

100% agreement.

The group agreed that fever should be avoided in individuals suffering from acute SAH, although the level of evidence for prophylactic normothermia is low Nevertheless, a discussion was held regarding the definition of body core temperature as it pertains to fever, as there is no established threshold for the occurrence of fever [48]. Our group concluded that fever should be treated when the core temperature exceeds 38 °C and that a target temperature should be held between 36.5 °C and 37.0 °C based on the association of fever and worsening of outcome as well as increase in vasospasm and DCI. In algorithms for fever treatment and control, the identification and treatment of potentially life-threatening infections must be included. Regardless of its cause, fever should be treated as soon as possible. Since fluctuations and increases in body temperature have been associated with poor outcome after stroke, worse modified Rankin Scale scores, and increased morbidity and mortality [49, 50], these should be avoided. Recently, a large clinical trial investigated fever prevention after acute vascular brain injury. Patients were randomized to fever prevention targeted to 37.0 °C for 14 days or intensive care unit discharge using an automated surface temperature management device. Standard care patients received standardized tiered fever treatment on occurrence of temperature of 38 °C or greater. As a result, preventive normothermia effectively reduced fever burden, but did not improve functional outcomes. Despite limited evidence, the group felt that preventive normothermia was important as part of a treatment bundle rather than a single intervention with major solitary impact. The importance of maintaining temperature at a consistent level was agreed upon by consensus.

“Consensus statement: Our expert group recommends fever prevention as an important part of the treatment after SAH due to the association of fever and worsening of outcome and increase in DCI and vasospasm despite low evidence.”

16. Temperature control should be done with an automatic TTM device in intubated patients with acute SAH if physical and/or pharmacological fever treatment fails. Side effects and risk–benefit ratio of automated TTM should be considered.

100% agreement.

If the body’s core temperature as a proxy for brain temperature exceeds 37.5 °C, our group intends to start TTM. However, as stated before evidence for this procedure is very poor.Since the data on automated TTM are rare and subject to debate, pharmacological and physical methods should be used first. Typically, paracetamol and metamizole are employed as medications, whereas cold infusions and calf cooling serve as examples of physical techniques. It is recommended that automated TTM devices include a feed-back driven console that measures body core temperature and adjusts cooling or heating to the desired target temperature. All methods for TTM should be outlined in a distinct protocol that specifies thresholds for fever treatment, methods for cooling, and the management of side effects. The group emphasizes the need to avoid and treat cold-induced shivering due to its potential negative effects [51–53]. It was agreed that to effectively manage shivering, it is imperative to incorporate counter-warming, paracetamol, sedatives, magnesium, opioids, and neuromuscular blocking agents into a protocol for temperature control. Such measures could be used in a stepwise approach, from non-sedating interventions to sedatives and neuromuscular blocking agents if first-line interventions are not successful. Protocols that have already been published could be used to create own clinical standards [54].

“Consensus statement: Our expert group recommends the use of semiautomated device for fever control, if the center is experienced its use and has a detailed SOP. However, the groups states the low level evidence of such TTM procedures..”

17. Experienced centres can use induced therapeutic hypothermia below 36 °C as a rescue therapy. This scenario can include vasospasm, DCI and ICP crisis. When using hypothermia, side effects should be avoided and closely monitored.

100% agreement.

Hypothermia can be considered a multimodal neuroprotective method [55]. Although animal data show clear benefits in focal and global cerebral ischemia and experimental SAH, clinical data are scarce [56]. Case series indicate beneficial effects on vasospasm, DCI, and functional outcomes. However, the interpretation of these data is limited by the low patient number, missing randomized control groups, and different hypothermia protocols [57–60]. Therapeutic hypothermia decreased the velocities measured in transcranial ultrasound, reflecting a temperature-dependent reduction of CBV and/or vasospasm [57–59]. It is theoretically possible that a decreased cerebral metabolic rate by temperature reduction would be beneficial in cases of critically limited blood and oxygen supply, such as vasospasm or DCI. In addition, ICP lowering effects of hypothermia has long been described [56]. Target temperature, duration, and rewarming rate are some of the major subjects of dosing hypothermia that have not been defined in SAH. Theoretical aspects include (A) a rapid reduction in target temperature to prevent critical focal ischemic events, (B) the duration of hypothermia for more than 24 hours, as the pathological trigger such as vasospasm does not fade rapidly, (C) avoiding temperature fluctuations, and (D) a slow rewarming process to prevent overshooting hyperthermia and rebound of the critical measures. All participants concurred that the administration of induced hypothermia should solely be accomplished through the utilization of automated feed-back devices and a comprehensive protocol. Since there is minimal evidence for such procedures, high level of experience on TTM and highly precise protocols are obligatory.

“Consensus statement: Our expert group recommends the use of TTM in critical situations such as ICP-crisis, severe vasospasm and DCI, if the center is experienced in its use and has a detailed SOP.”

### Management of hydrocephalus

18. Management of external ventricular and lumbar drainage.
19. **CSF drainage via external ventricular drainage should be carried out continuously. Agreement 56% (9 votes in favor, 5 abstentions, 2 reject).**
20. **CSF drainage via external ventricular drainage should be carried out on-demand (ICP-dependent). 43% agreement (8 abstentions, 1 vote against).**

Currently, there are no recommendations available for the management and timing of CSF-drainage after aSAH. The DIVI Study group provided no valid recommendation regarding extraventricular (EVD) drainage management, e.g. continuously vs. intermittent on-demand drainage volume of external ventricular drainage. Both drainage types were stated as favorable. The idea behind intermittent drainage is that continuous drainage with a low intracranial pressure (ICP) might not support the pressure gradient required to re-establish the natural pathways for cerebrospinal fluid (CSF) outflow. Continuous external ventricular draininage (EVDs) have been linked to more complications related to intermitted EVDs [37, 38]. Evidence on other outcomes is mixed. Some retrospective studies indicate that continuous drainage could lead to higher rates of delayed cerebral ischemia (DCI) and ventriculoperitoneal shunt (VPS) placements [39], however, there are also data, suggesting effective CSF drainage may reduce the risk of vasospasm, delayed ischemic neurological deficits, and poor outcomes [60]. To clarify these conflicting findings, a single-center randomized trial was conducted to compare vasospasm rates between continuous and intermittent EVD management strategies [61]. The study found no significant difference in the primary outcome of vasospasm, although there was a non-significant increase in angiographic vasospasm in the intermittent group (35% vs. 21%). Importantly, the study was halted early by the Data Safety and Monitoring Board because interim analysis showed a significant rise in EVD complications in the continuous drainage group. The 53% vs. 23% difference in complications was mainly due to more frequent EVD clogging in the continuous group. Additionally, the continuous group experienced a higher, though not statistically significant, rate of ventriculitis, with rates much higher than those reported in other studies [62].

(iii) CSF drainage volume via external ventricular drain should be carried out with a fixed volume.

33% agreement (2 abstentions, 8 votes against).

The evidence of a hydrocephalus is a common complication after an aneurysmal subarachnoid hemorrhage, which is often treated by insertion of an external ventricular drain. However, the management of CSF drainage volume is discussed controversially and performed with a wide range in neurointensive care units. The volume of CSF drainage is a frequently used parameter to treat increased ICP. However, increased CSF drain volume affects the risk of permanent shunt dependency in a negative manner, so that several departments used a fixed CSF drain volume. Currently there are no valid recommendations with respect to CSF drain volume available. The DIVI Study group made no specific recommendations regarding the drainage volume of external ventricular drainage, although several members reject a fixed drainage volume.

“Consensus statement: Our expert group does not recommend fixed volume drainage management in EVD:”

(d) Lumbar drainage can be used for improvement of clearance for the CSF space and weaning from the EVD.

100% agreement.

Lumbar drainage of CSF after aSAH has been shown to reduce the prevalence of DCI and improve early clinical outcome [63]. ICP monitoring may be considered in patients with suspected intracranial hypertension even in the absence of hydrocephalus. The placement of an EVD or lumbar drain can be determined by hemorrhage or hydrocephalus CSF flow pattern. In terms of timing of EVD, no data exist. However, in centers without expertise in aneurysm treatment, urgent stabilization of the patient, CSF diversion, and EVD placement, if needed, should be performed before transfer to a an aSAH treating center is established.

“Consensus statement: Our expert panel recommends that lumbar drainage can be used for improving the clearance of the CSF from blood and EVD weaning in experienced centers.”

### Detection and treatment of DCI and vasospasm

19. Transcranial ultrasound should be used to detect and monitor early vasospasm.

93% agreement (1 vote against).

As safe and non-invasive bedside monitoring technique TCD allows repetitive and dynamic assessment of vasospasms after SAH. The evidence of vasospasm in TCD was found to be highly predictive of DCI [64]. Additionally, evidence of vasospasm correlates strongly with cerebral infarction after aSAH [65]. The pooled estimates for TCD diagnosis of vasospasm (for DCI) had a sensitivity of 90% (95% CI, 77%–96%), a specificity of 71% (95% CI, 51%–84%), a positive predictive value of 57% (95% CI, 38%–71%), and a negative predictive value of 92% (95% CI, 83%–96%). Advantages of the method are ubiquitous availability, the avoidance of transportation of critically ill patients and unlimited repeatability. Limitations are the operator dependence, patient’s anatomy, affection by physiological measures (BP, Heart rate) and only intermittently performance [66, 67]. Nevertheless, in the opinion of the authors, it is a valuable screening method especially for patients, where neurological assessment is not available. Since the duplex flow velocity measurements are always only a snapshot several examinations a day can help to detect flow velocity changes early.

“Consensus statement: Our expert panel recommends the use of TCD for screening and monitoring of vasospasm.”

20. Nimodipine should be used routinely from the beginning of treatment.

100% agreement.

Continued enteral administration (60 mg 6 times a day) proved beneficial in preventing DCI and improving functional outcome after aSAH. This was originally published in 1983 [68] and thereafter confirmed by a meta-analysis of 16 trials involving 3361 patients [69]. Because interruption of nimodipine administration has been shown to be associated with a higher incidence of DCI [70], consistent administration from the beginning is recommended. The optimal management of nimodipine therapy (dose reduction or discontinuation) in patients experiencing relevant hypotension remains unclear.

“Consensus statement: Our expert panel recommends the early use of nimodipine for prevention and treatment of vasospasm.”

21. Intravenous nimodipine can be administered as an alternative to enteral nimodipine, considering the side effects.

100% agreement.

Enteral and intravenous nimodipine showed similar efficacy in preventing poor outcome, delayed cerebral ischemia and ischemic neurologic deficits. This was reported by two recent meta-analyses that included up to ten studies with a total of 1527 randomized patients [71, 72]. However, the validity of existing studies is limited due to risk of bias, so future high-quality RCTs are needed to make stronger recommendations for routes of nimodipine administration [71, 72]. Side effects, particularly a greater incidence of hypotension, pulmonary edema and renal insufficiency associated with intravenous nimodipine, should be considered [73].

“Consensus statement: The expert panel recommends that intravenous nimodipine can be used as an alternative to enteral nimodipine.”

22. In patients who can be examined appropriately with suspected DCI, cerebral imaging via CT or MRI with perfusion should be performed.

66%agreement (5 abstentions)

CT-P or MR-P helps to identify alterations to the microcirculation, in addition to the macroscopic vasospasm that can be detected with CTA. In patients who show new focal clinical signs which might be attributed to DCI, CT-P or MR-P should be done.

“Consensus statement: The expert panel could not agree on the routine use of CT- or MR-perfusion in patients who can be examined appropriately.”

23. In patients with suspected vasospasm cerebral or DCI who cannot be examined appropriately imaging via CT or MRI including perfusion imaging should be performed.

100% agreement.

Monitoring for vasospasm and delayed cerebral ischemia (DCI) presents challenges in clinically not assessable patients. However, surrogate parameters such as flow velocity in cerebral arteries measured on transcranial Doppler (TCD), continuous EEG monitoring, or various metabolic markers (such as microdialysis) can be utilized. Assessing the risk of permanent brain damage is crucial for determining the appropriate therapeutic approach. Even in the absence of vasospasm on angiography, perfusion imaging may reveal focal hypoperfusion. Positive findings on perfusion studies are shown to be associated with a significantly higher likelihood of DCI. [74–77].

MRI perfusion can also identify the tissue at risk, prompting consideration of invasive angiographic interventions [78].

“Consensus statement: The consensus among authors taht perfusion imaging should be used in patients with suspected vasospasm or DCI who cannot be clinically evaluated, aiding in the determination of the most suitable therapeutic course.”

24. Induced hypertension should be performed if DCI is detected.

100% agreement.

There is only one RCT on induced hypertension in aSAH, the HIMALAIA trial (Hypertension Induction in the Management of Aneurysmal Subarachnoid Haemorrhage With Secondary Ischaemia), which was prematurely halted due to missing effects on cerebral perfusion and slow recruitment. There was no difference in functional outcome in this underpowered trial [79]. However, observational data from large cohort studies suggest neurological improvement after induced hypertension in about 80% of symptomatic patients [79–81]. Existing data therefore suggest that clinically established induced hypertension may be useful in patients with DCI.

“Consensus statement: The consensus group recommends that induced hypertension should be used in patients with DCI.”

25. After the diagnosis of DCI and in the absence of contraindications, the MAP should be raised to at least 80 mmHg, in individual cases even higher.

100% agreement.

As the clinical evidence for induced hypertension is limited, the recommendation of a minimum value for MAP is mainly based on pathophysiological considerations regarding impaired cerebral perfusion, especially in patients with elevated ICP and patients with DCI, and the observation that blood pressure variability is associated with less favorable neurological outcomes [82]. In the HIMALAIA trial patients were treated with fluids and norepinephrine to raise the MAP up to a maximum of 130mmH [79]. In the control group, a minimum MAP of 80 mmHg was maintained with fluids and, if necessary, with vasopressors. According to these data the authors recommend a lower limit of MAP of 80 mmHg with individually induced hypertension above in presence of DCI. Goal-directed therapy including multiparametric advanced hemodynamic monitoring, which indicates continuous cardiac output (CCO), (volumetric) preload, fluid responsiveness, and extravascular lung water can save vasopressors and appears to prevent DCI in a monocentric study [31].

“Consensus statement: The consensus group recommends to maintain a MAP of at least 80mmHg and the use of multiparametric advanced hemodynamic monitoring in case of DCI. ”

26. Interventional rescue therapy should be done in DCI 94% agreement (1 abstention).

Interventional treatment including spasmolysis and angioplasty are available in case of vasospasm and DCI. These approaches can be used as first line or if other therapeutic approaches such as induced hypertension fail. Because efficacy has not been proven so far in a randomized approach, benefit of interventional treatment for DCI prevention/reversal is controversial [1].

“Consensus statement: The consensus group recommends that interventional treatment should be as a rescue therapy.”

27. Angioplasty is an opportunity for the treatment of severe vasospasm and DCI if pharmacological spasmolysis is not successful.

100% agreement.

Despite conservative strategies to improve cerebral perfusion and the use of intravenous or oral vasodilating agents, severe cerebral vasospasm (> 50% reduction in the diameter of the affected vessel) may still occur, potentially leading to delayed cerebral ischemia (DCI) and poor outcomes. In such cases, selective catheterization of the affected artery with intra-arterial injection of vasodilating agents and/or angioplasty can be performed. However, the effect of intra-arterial injection is transient, necessitating repeated administrations [83, 84]. Angioplasty can be performed using various devices, including compliant or non-compliant balloons, stent retrievers, expandable and removable stent-like devices (e.g., Comaneci, Cascade), or dedicated retrievable stents (e.g., pRELAX) [83–86]. When using stent retrievers or pRELAX, the device is temporarily deployed in the affected vessel segment and removed after 3 to 5 min [83, 85, 86]. In balloon or Comaneci-assisted angioplasty, a balloon is inflated, or the Comaneci device is gradually expanded in the diseased segment for a shorter duration and then deflated or relaxed [83, 84, 87]. Both techniques are highly effective, safe, and provide immediate, long-lasting relief from vasospasm, with low recurrence rates compared to intra-arterial infusion therapy [83, 84]. While intra-arterial administration of vasodilating agents affects the entire arterial circulation, regardless of vessel size, advancements in device technology now allow balloon catheters to be used for treating distal cerebral vasculature spasms as well [86]. Additionally, improving the calibre of proximal vessels can have a cascading effect, reducing vasospasm in the distal vasculature [83, 84].

“Consensus statement: Our group recommends the use of stent- or balloon-assisted angioplasty, either alone or in combination with intra-arterial infusion therapy, as an opportunity in patients with medically refractory severe vasospasm, to prevent DCI and improve clinical outcomes.”

### Seizures and EEG monitoring

28. If a seizure is in question, anticonvulsive medication should be given but reevaluated carefully after the acute phase

100% agreement.

An important aspect in the treatment of SAH patients are seizures, which occur with an incidence of 4–20% in patients with intracerebral hemorrhage [88–90]. Seizures mostly occur within the first three days after the onset, with a lobar and subcortical bleeding location associated with an increased risk. However, the occurrence of seizures is not associated with a worsened outcome after 6 months [89–93]. Furthermore, several studies have shown the negative impact of prophylactic anticonvulsive medication (ACM) on neurological outcomes, so an individualized ACM should only be initiated after the occurrence of a seizure [91–97]. The need for ACM should be critically examined over time, as less than 15% of patients develop structural epilepsy [92–97].

“Consensus statement: Our group recommends the use anticonvulsive medication in clinically suspected seizures and later revaluation.”

29. A prophylatic anticonvulsive therapy might be considered in patients with intracerebral hematoma.

0% agreement (all votes against).

Seizures are a common complication in aSAH patients. AHA/ASA- guidelines recommend an anticonvulsive treatment in patients with an seizure at aSAH onset for ≤ 7 days [1]. Additionally, in patients with high-seizure-risk features (e.g. ICH, ruptured MCA-aneurysm, high grade SAH) a prophylactic anticonvulsive treatment may be considered [98]. German guidelines suggest an anticonvulsive treatment in the acute phase without long-term medication [99].

Until now there is no proof of benefit for a prophylactic anticonvulsive treatment in aSAH patients although seizures at aSAH onset are predictive for a poor outcome [100, 101]. Seizures with later onset – after more than 24 h—in aSAH patients are not an outcome predictor and prophylactic treatment does not prevent such late onset seizures [88, 102].

“Consensus statement: The panel voted 100% against a prophylactic anticonvulsive treatment. Early onset seizures should be treated for at least 1 week, potentially longer if necessary.”

30. In patients difficult to assess neurologically an EEG monitoring (if applicable continuously) is a rational option for monitoring.

100% agreement.

Clinical data indicate that seizures occur in up to 28 percent after aSAH [101, 102]. Recent data indicate electrographic seizures in patients with aSAH in up to 17.6% of patients monitored expressly because of clinically suspected subclinical seizures [102]. In unselected patients, seizures still occurred in 9.6% of all cases, and in 8.6% of cases in which there was no a priori suspicion of seizures. As many patients require sedation and mechanical ventilation, seizures are difficult to detect on clinical examination.

“Consensus statement: Our consensus group recommends the use of EEG for patients in whom examination is difficult due to their state of consciousness.”

31. In patients with intracerebral hematoma, routine EEG diagnostics should be performed to detect and treat possible seizures. The duration of the anticonvulsive medication should be based on clinical criteria and EEG diagnostics.

100% agreement.

The occurrence of seizures is described in up to 25% of patients with SAB, especially with accompanying intracerebral hemorrhage (ICH) [88–90]. These seizures often occur non-convulsive and are therefore underdiagnosed [89]. Using continuous EEG monitoring, epileptic activity could be demonstrated in about 20% of comatose patients even 2 weeks after acute subarachnoid hemorrhage [90]. The application of continuous or discontinuous EEG measurement as a non-invasive method for neuromonitoring is to recommend to detect and treat non-convulsive seizures [91].

“Consensus statement: Our expert group recommends the frequent use of EEG in patients with ICH and adaption of anticonvulsive treatment based on clinical criteria and EEG.”

32. The routine use of antiplatelet agents to prevent DCI is not recommended.

100% agreement.

In accordance with the 2023 AHA/ASA guideline, neither the administration of antiplatelet nor anticoagulation can be endorsed or discouraged due to insufficient or conflicting evidence regarding their efficacy in preventing DCI [1]. In the systematic review conducted by Mees et al. on use of antiplatelet therapy (APT) for aSAH, a tendency toward improved outcome in patients treated with antiplatelet agents was observed, likely attributed to a reduction in secondary ischemia [103]. However, these findings did not reach statistical significance, thus precluding definitive conclusions. Among those studies, antiplatelet regimens exhibited considerable variability. Additionally, the use of antiplatelet agents might elevate the risk of hemorrhagic complications. A recent review and meta-analysis included 14 studies with 4228 aSAH patients. APT after aSAH was associated with good functional outcome, but there was no relationship with delayed cerebral ischemia or intracranial hemorrhage [104]. Based on the existing evidence, our expert group unanimously advises against the routine use of antiplatelet drugs for DCI prevention.

“Consensus statement: Our panel does not recommend the routine use of antiplatelet agents to prevent DCI in aSAH to prevent DCI.”

### Invasive monitoring

33. In cases where patients are not assessable, multimodal monitoring can be used for better therapy control. This includes parenchymal oxygen partial pressure measurement (PtiO2), jugular venous oxygen saturation, NIRS, microdialysis, and/or continuous EEG.

100% agreement.

Currently, ICP and CPP monitoring are regarded as the fundamental procedures of neuromonitoring for patients who cannot be assessed or who are sedated. Furthermore, there are several options for extending multimodal monitoring. The primary source of energy for the human brain is derived from aerobic metabolism. Therefore, given the limited cerebral reserves of oxygen and adenosine triphosphate, it is imperative to maintain a consistent and adequate supply of nutrients and oxygen [105–112]. However, using only basic neuromonitoring with ICP and CPP, it is not possible to make any statements about the brain’s oxygen supply or metabolic situation [106–109]. Due to these limitations, additional diagnostic methods have been developed for monitoring cerebral oxygenation and metabolic status [1–8].

At present, non-invasive near-infrared spectroscopy (NIRS) and invasive methods of jugular venous oximetry and oxygen partial pressure measurement in brain tissue are available for assessing cerebral oxygenation [105–112]. The use of jugular vein oxygen saturation in the internal jugular vein allows for a statement about global cerebral oxygen consumption. The normal range for SjvO2 is given with a range between 54 and 75%. Schneider and colleagues report that the SjvO2 values below 50% have a negative impact on the outcome [105–112]. However, due to the high susceptibility to artifacts and the significant time and personnel resources required, this method is mainly reserved for specific scientific-experimental questions. Another method of objectifying cerebral oxygenation is the direct measurement of oxygen partial pressure in brain tissue (ptiO2) using an implanted measuring catheter [105–112]. PtiO2 normal values are about 22 mmHg in the white matter and increase to approximately 33 mmHg in the cortex [106–112]. PtiO2 values < 10 mmHg are considered critical, so an intervention recommendation exists for ptiO2 values < 15 mmHg [106–112]. Due to the restricted measuring radius of approximately 17 cubic millimeters, the positioning of the ptiO2 probe holds significant significance. It is possible that ptiO2 values will be lower in intra- or perilesional locations than in vital brain tissue, so the course of ptiO2 measurement values will play a decisive role. In addition to the possibility of continuous ptiO2 measurement, the low incidence of procedure-associated complications pertaining to ptiO2 probe placement, infections, and bleeding is noteworthy. The disadvantages of this method include its regional measuring character, the invasiveness of the procedure, and the divergence of physiological and pathological measurement values between the available probe systems. This complicates the direct transfer of measurement values. Due to these limitations, ptiO2 measurement is not extensively utilized in neurosurgical intensive care medicine. Near-infrared spectroscopy (NIRS) allows for the monitoring of cerebral perfusion and oxygenation as a non-invasive and bedside-available method [106, 107, 112]. Based on NIRS technology, it is possible to detect alterations in regional CBF and cerebral oxygenation by measuring the concentration changes of oxyhemoglobin and deoxyhemoglobin. Due to its high susceptibility to disturbances with an increase in extracranial blood (e.g. hematomas), sweating on the skin, subdural/epidural blood or air inclusions, motion artifacts, air inclusions between the sensor and skin, or sensor placement over the frontal sinus, this technique has not yet found its way into the clinical routine of neurosurgical intensive care medicine. 

Cerebral microdialysis allows the local measurement of neurotransmitters (glutamate, glycerol) and metabolites (glucose, lactate, pyruvate) in the brain tissue’s interstitial fluid, providing information about cerebral metabolism [112–115]. A microdialysis catheter is implanted either as part of surgical care or through a drill hole trepanation. After the placement of the microdialysis catheter, it is imperative to evaluate its position using cranial computed tomography to ensure the accurate interpretation of the measurement data. There are fundamentally different measurement values expected in areas of vital brain tissue compared to areas of contusion or vasospastic tissue [112–114].

Numerous studies have established normative values for microdialysis parameters. Because of the strong location dependence and the limited spatial measurement area of the microdialysis catheter, both the absolute values and the course of the measurement values should be included in further therapy planning [113, 114]. Microdialysis can be fundamentally used for the early detection of cerebral ischemia, the control of therapeutic measures, and the prognosis determination. The disadvantages of the method include its invasiveness, the high personnel effort required, the need for imaging position control, the location dependence in brain tissue, and the lack of the possibility of a global view of cerebral metabolism [113, 114]. Due to their high susceptibility to disturbances, extensive personnel requirements, and high expenses, these methodologies, particularly in light of the highly heterogeneous study environment, have not yet been incorporated into routine clinical practice.

“Consensus statement: The use of extended multimodal monitoring can be used as additive methods for controlling intensive medical therapy if established in the facility.”

34. In the presence of reliable pathological findings (ptiO2 < 15 mmHg) exclusively in the ptiO2 measurement, a therapeutic attempt should be made by increasing the CPP and/or raising the inspiratory O2 concentration. Other patient`s parameters should be interpreted in conjunction with the ptiO2 value.

100% agreement.

When pathological ptiO2 levels are < 15 mmHg, there is a risk of local ischemia, so the supply of oxygen and nutrients, especially glucose, should be increased. By raising the CPP or increasing the oxygen supply, more oxygen or glucose can be provided to the human brain to prevent ischemia [105–110]. However, the extent to which the brain can utilize the additional substrates is uncertain [105–108]. For measuring utilization, the additive use of cerebral microdialysis could be considered in this case. Considering the known limitations of a local ptiO2 measurement, raising the CPP and/or the inspiratory oxygen fraction appears to be the only effective measures to prevent ischemia, so this therapeutic attempt should be made [105–107]. Another option to improve ptiO2 is using the CPP_opt_, if the technological standard is available (100). However, ptiO2 measurements and therapy according to critical values has not been shown to improve clinical outcome, so far. Therefore, this technique should be used with caution and under consideration of the patient’s clinical condition and other parameters.

“Consensus statement: The panel recommends therapeutic interventions when brain tissue oxygen monitoring indicates critical values in conjunction with other parameters and the clinical condition of the patient.”

## Discussion

The consensus statement presented in this manuscript provides a pragmatic approach to the acute management of aneurysmal subarachnoid hemorrhage (aSAH), addressing critical areas where evidence is either limited or inconsistently applied in clinical practice. The inclusion of a multidisciplinary expert group highlights the importance of integrating multiple perspectives from neurology, neurocritical care, neurosurgery, and interventional neuroradiology to create robust and practical guidelines. Certainly, practical experience based on successful and usable local institutional protocols influenced the consensus statements. Therefore, our results can be interpreted as the multiinstitutional protocol with a target audience of treating physicians looking for guidance on issues not covered by other evidence-based protocols.

In consequence, thisconsensus focuses on both evidence-supported strategies and areas where clinical expertise may guide decision-making. For example, while high-grade recommendations exist for early aneurysm treatment and blood pressure management, other domains—such as therapeutic hypothermia or multimodal monitoring—are characterized by low evidence levels but high clinical relevance. By emphasizing standardized protocols for such interventions, the consensus statement aims to harmonize care delivery and reduce variability across treatment centers.

Moreover, the consensus addresses emerging technologies and methodologies, such as continuous temperature management, advanced imaging for vasospasm detection, and multimodal monitoring. These practices underscore the potential for innovation to improve patient outcomes but also highlight the need for further research to validate their effectiveness.

Despite the comprehensive nature of this consensus, certain limitations warrant discussion. The reliance on expert opinion in areas lacking robust evidence introduces a degree of subjectivity, and the recommendations may not fully account for regional variations in resource availability or institutional expertise. Additionally, while the modified Delphi process ensured broad agreement, it is inherently limited by the perspectives and experiences of its participants. An imbalance in different subspecialties and selection of authors could be criticised methodologically and may have had impact on the consensus statements which is a risk of bias in such projects.However, except for the neuroradiologists, all subspecialties have extensive experience in neurocritical careTherefore, we reach a rather homogenous neurocritical care experience level. As seen in the voting, a more balanced group would not have changed the vast majority results in the consensus statements. It could be argued that sponsoring of the first personal meeting biased the discussion and results. However, all participants of the consensus process were selected on behalf of the speaker of the DIVI section after discussion within the section.

The consensus aligns well with recent updates in international guidelines, such as those from the American Heart Association (AHA), reinforcing global best practices while adapting them to the specific needs and capabilities of German-speaking regions. This regional focus may limit the direct applicability of the guidelines to other healthcare settings but serves as a valuable model for consensus-driven guideline development and practical implementation. Moreover, it will improve the harmonisation of protocols of different specialities of medicine such as anaesthesiology and neurology departments who take the main responsibility for the patient.

## Conclusion

This multidisciplinary consensus statement provides a practical framework for the acute management of aSAH, bridging gaps in evidence and offering actionable recommendations for multiple clinical scenarios. By integrating expert opinion with available evidence, these guidelines aim to standardize care, improve patient outcomes, and identify key areas for future research. Adoption of these consensus statements will depend on their validation in clinical practice and their alignment with institutional resources and capabilities. Further studies, particularly randomized controlled trials, are needed to address the evidence gaps identified in this consensus and to refine the guidelines for broader applicability. 

## Data Availability

Written data provided by authors.
